# Kinetics of lactose hydrolysis and galactooligosaccharides formation in beverages based on goat’s milk and its permeate

**DOI:** 10.1007/s10068-019-00600-0

**Published:** 2019-04-06

**Authors:** Łukasz K. Kaczyński, Dorota Cais-Sokolińska, Artur Szwengiel

**Affiliations:** 1 Department of Dairy Products Quality, Faculty of Food Science and Nutrition, Poznań University of Life Sciences, ul. Wojska Polskiego 31, 60-624 Poznan, Poland; 2 Department of Fermentation and Biosynthesis, Faculty of Food Science and Nutrition, Poznań University of Life Sciences, ul. Wojska Polskiego 31, 60-624 Poznan, Poland

**Keywords:** Galactooligosaccharides, GOS, Goat milk, Lactose, Permeate

## Abstract

The aim of this work was the analysis of galactooligosaccharides (GOS) formation in a model mixture of goat’s milk and its permeate from microfiltration and further concentration by ultrafiltration based on the hydrolysis and transgalactosylation of lactose under various temperature and time regimes. These reactions were catalyzed by a β-galactosidase from *Kluyveromyces lactis.* Simultaneous hydrolysis and transgalactosylation of the milk lactose was carried out at 37, 40, and 43 °C for 6 h. The maximum GOS content in the mixture was obtained at 37 °C after 20 min. It was 6.9% of the total sugars and the degree of lactose hydrolysis was 13.3%. This was about 10% more GOS than in milk. The mixture containing GOS had a faster maximum acidification rate, 33% greater than before transgalactosylation.

## Introduction

Goat’s milk, due to its unique characteristics is of particular interest to consumers and producers and can be the basis for the creation of new dairy products using enzymatic processes and membrane separation (Cais-Sokolińska et al., 2015; Pikul et al., 2014). For example, milk permeate can be subjected to the same technological processes as milk, e.g. lactose hydrolysis, which leads to a further reduction in the lactose content of the finished product.

The lactose content in goat’s milk is about 4.7% and is slightly higher than in cow’s milk. Lactose in milk may be a substrate in multidimensional chemistries, including hydrolysis, fructosyl transfer, transgalactosylation, isomerization, oxidation, and reduction. Such processes may involve glucose, galactose, lactosucrose, galactooligosaccharides (GOS), lactulose, lactobionic acid, and lactic acid (Santibáñez et al., 2016).

GOS are complex mixtures consisting of galactose and glucose, with a molecular (Gal)_n_–Glu structure. They belong to so-called prebiotics, which are non-digestible and show many beneficial functions in the human body (Córdova et al., 2017). Galactooligosaccharides are the products of lactose transgalactosylation reactions, along with other structurally related galactosides. The GOS synthesis reaction is catalyzed by glycosidases, including β-galactosidases. GOS after the transgalactosylation process consist of oligosaccharides with varying degrees of polymerization, significant amounts of monosaccharides (glucose and galactose) and unreacted lactose. The removal of sugar compounds (monosaccharides and lactose) from raw GOS is a fundamental requirement for the introduction of GOS into functional foods (Córdova et al., 2017; Maischberger et al., 2008).

In addition to using commercial preparations of GOS they can be produced by a controlled process from an appropriate choice of raw material, such as milk or its mixture with another raw material. This knowledge provides the basis for the search for raw materials other than milk, especially those that are treated as by-products, as a source of GOS synthesis. For example, the cheese production process is preceded by the fractionation of milk by microfiltration (MF) to increase yield by increasing the proportion of casein and eliminating part of the whey protein. The resulting MF permeate, which contains native whey proteins (serum proteins), is an example of such a material.

The main aim of the study was the analysis of GOS formation in a model mixture of goat’s milk and its permeate from MF and further concentration by ultrafiltration (UF) based on the simultaneous hydrolysis and transgalactosylation of lactose under various temperature and time regimes. Such processes give rise to the ability to improve the functionality and management of the MF/UF permeate from goat’s milk. The mixture is intended to be the raw material for the production of an innovative kefir made using milk-alcohol fermentation.

## Materials and methods

### Materials

The goat’s milk used from Polish White Improved goats from farms located in the Wielkopolska region (Western Poland). The skimmed milk was subjected to MF using ceramic ISOFLUX™ membranes made of ZrO_2_–TiO_2_ and produced by Tami Industries (Nyons, France). Membranes of 1.4 μm pore size were used to reduce the number of microorganisms.

### MF/UF permeate

For the second MF of the milk, membranes of 0.14 μm pore size were used to aid protein separation. These were the membrane 23 channels with a channel diameter Ø = 3.6 mm, an outside diameter Ø = 25 mm, a length of 1 178 mm (Tami Industries, Nyons, France). The feed stream was fed to the interior of the tube in cross-flow with a rate of 4.0–4.5 m/s. The transmembrane pressure during microfiltration totaled 0.5–0.8 bars. The obtained permeate was subjected to further compaction during the ultrafiltration process (UF). Here, ceramic membranes of Nominal Molecular Weight Cut-Off of 30 kDa (CeRAM INSIDE ISOFLUX™ Tami Industries, Nyons, France) were used. The UF was carried out to achieve a 4.5–5.0-fold increase in the level of solid non fat similar to goat’s milk (85 g/kg).

### Mixture of goat’s milk and MF/UF permeate

Goat’s milk and MF/UF permeate were combined in a ratio of 60:40 (%, v/v). The combination of goat’s milk with MF/UF permeate in a ratio of 60:40 (%, v/v) allowed for the alteration of the ratio of casein to whey protein from 4.2 in goat’s milk to 1.1 when milk was mixed with permeate in the above proportion. It was assumed that this is a ratio at which the ratio of lactose (L) to protein (TP) is at an intermediate level between goat’s milk (1.3) and the permeate (1.6), giving an L:TP ratio of 1.4. The mixture of milk and the permeate was kept in a chilled state at 3 ± 0.5 °C for up to 2 h.

### Enzymatic conversion of lactose into galactooligosaccharides

These reactions were catalyzed by a β-galactosidase from *Kluyveromyces lactis* named GODO–YNL2 (DuPont™ Danisco A/S, Brabrand, Denmark). The enzyme (5 000 U/g) was added at a concentration of 0.1% (w/v). Simultaneous hydrolysis and transgalactosylation of the milk lactose was carried out at 37, 40, and 43 °C for 6 h. The samples were then pasteurized at 92 °C for 15 min in order to inactivate the enzyme.

### Fermentation

The pasteurized milk, permeate, and their mixture after hydrolysis and transgalactosylation of lactose were fermented in a lacto-alcoholic fermentation process using a mixture of mesophilic strains of lactic acid bacteria (LAB) and yeast from Abiasa Inc.’s (Quebec, Canada) collection (code of 75106, 30 u.a. 100 1/L of milk). Fermentation was at a of 22 °C. The dose of introduced cultures was selected so that the end point of the fermentation would be a product with a pH value of 4.4.

### Compositional analysis

Determination of the basic chemical composition was conducted using standard methods (AOAC, 1995; AOAC, 2000).

### Determination of glucose, galactose, lactose and galactooligosaccharides (GOS)

The GOS composition was determined according to AOAC method 2001.02, using LC–MS instead of HPAE–PAD to detect galactose, glucose, and lactose (Slegte, 2002). The degree of lactose hydrolysis (DLH) was calculated from the formula Eq. (1) (Adamczak et al., 2009):

1 $${\text{DLH}} = \left( {1 - \frac{RL}{IL}} \right) \times 100\%$$

where RL is residual lactose concentration (g/kg), IL is initial lactose concentration (g/kg).

Kinetics of formation of galactooligosaccharides was the relationship between GOS content in total sugars during the 6-h reaction. In the first hour of the reaction, an assay was carried out every 20 min, and then every hour thereafter.

### LC–MS analysis

An ion-exclusion high-performance liquid chromatography electrospray ionization mass spectrometry (IEHPLC–ESI–MS) analysis was performed using a Dionex UltiMate 3000 UHPLC (Thermo Fisher Scientific, Sunnyvale, CA, USA). He was coupled to a Bruker maXis impact ultra-high resolution orthogonal quadrupole-time-of-flight accelerator (qTOF) equipped with an ESI source and operated in positive ion mode (Bruker Daltonik, Bremen, Germany). The IE chromatographic separation was achieved with a Rezex™ RCM-Monosaccharide Ca^2+^ (8%), 300 × 7.8 mm LC Column (Phenomenex, Torrance, CA, USA). The separation and detection parameters were reported previously in detail by Gumienna et al. (2016). d-glucose-1-^13^C and D-galactose-1-^13^C were used as internal standards. An external calibration was performed for lactose.

### Kinetics of coagulation

The pH was measured using a CP-502 pH meter (Elmetron, Zabrze, Poland) with ES AgP-301W (Eurosensor, Gliwice, Poland). The maximum acidification rate (V_m_) was calculated from the pH curves according to the equation V_m_ = (ΔpH/Δt) and expressed in absolute values (units of pH/h) (Kristo et al., 2003).

### Statistical evaluation

To verify the statistical hypotheses, a univariate ANOVA was employed, a level of significance of α = 0.05 was adopted, were performed using Statistica data analysis software version 10 (StatSoft, Inc. 2011).

## Results and discussion

### Composition of unfermented goat’s milk, permeate, and their mixture

It was demonstrated that the goat’s milk contained 32.0 g/kg protein and 41.4 g/kg lactose. This composition was typical of goat’s milk and coincided with the literature (Bruzantin et al., 2016; Serhan et al., 2016). The permeate was different from the milk, both qualitatively and quantitatively. Despite the removal of casein, the permeate contained only 14% less protein than the raw material. The MF/UF permeate had a protein level of 27.4 g/kg (*p *> 0.05; Table 1). The share of whey proteins in the total protein was 97%. The content of whey protein in the permeate (26.7 g/kg) was 4.3 times higher than in milk (*p* < 0.05), while no differences in lactose content were observed between milk and permeate (*p* > 0.05). The permeate after MF was destitute of casein, and a very similar proportion of whey protein in the total protein (96%) was previously noted (Svanborg et al., 2014).

**Table 1:** Content of ingredients in goat’s milk, permeate, and their mixture (6:4), *n *= 9, ± SD

	Milk	Permeate	Mixture
SNF (g/kg)	85.21 ± 0.70a	84.37 ± 1.20a	85.06 ± 0.80a
F (g/kg)	0.04 ± 0.01a	0.01 ± 0.01a	0.02 ± 0.01a
TP (g/kg)	32.00 ± 0.40b	27.40 ± 0.20a	30.30 ± 0.10b
CN (g/kg)	25.80 ± 0.10c	0.10 ± 0.20a	15.40 ± 0.30b
WP (g/kg)	6.20 ± 0.10a	26.70 ± 0.10c	14.00 ± 0.10b
CN:WP	4.20	< 0.004	1.10
L (g/kg)	41.40 ± 0.70a	43.10 ± 0.40a	42.20 ± 0.20a
L:TP	1.30	1.60	1.40

*SNF* solid non-fat, *F* fat, *TP* total protein, *CN* casein, *WP* whey protein, *L* lactose, *SD* standard deviation. a–c, different letters with mean values in row indicate statistically significant differences at the level α = 0.05

### Enzymatic hydrolysis and transgalactosylation of lactose

**Fig. 1:**
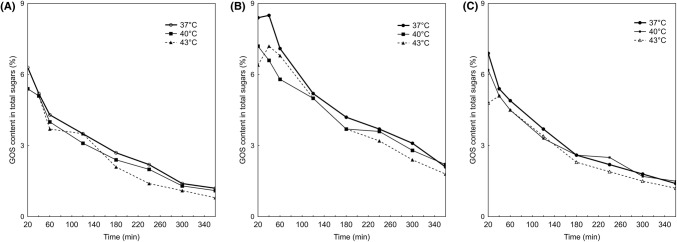
Kinetics of GOS synthesis in the enzymatic hydrolysis and transgalactosylation reaction of lactose in: (**A**) goat’s milk; (**B**) permeate; (**C**) its mixture, 60:40

The enzymatic bioconversion of lactose was induced in this reaction mixture. Figure 1 showed the amount of GOS produced was higher in the permeate than in the milk (*p* < 0.05). It was observed that the largest amounts of GOS in the mixture were formed during the first hour of reaction. After 20 min of reaction, the content of GOS in the mixture of milk and permeate was on average 5.9 ± 0.9% of the total sugars, and after 6 h reaction was on average 1.4 ± 0.1% of the total sugars. These were average values 29% less than in the permeate itself (*p* < 0.05). No differences were found between the amount of GOS produced in milk and the mixture at a temperature of 40 °C, except for the first 20 min of reaction (*p* < 0.05). The quantity produced in milk and in the mixture at 40 °C after 40 min was the same, and represented 1.5% of the total sugars.

It is generally accepted that the production of GOS grows with increasing lactose concentration. It is related to the decreased availability of water as an alternative galactosyl acceptor. Also, the use of organic solvents instead of water in the process favors transgalactosylation. Although the reduction in water activity by organic solvents improves GOS production, this effect is not entirely linear (Gänzle, 2012).

The factors that affect GOS production are, in addition to the amount of lactose, also the ions present in the lactose solutions, e.g. the amount and type of anions or cations in the permeate or whey milk. As reported by Fischer and Kleinschmidt (2015), the level of GOS production was 4.3% in sweet whey and 10.6% in acid whey. Ions may act as activators or inhibitors, depending on the origin of the enzyme.

The productivity of the enzymatic process of transgalactosylation depends not only on the initial concentration of lactose, but also on temperature, reaction time, water activity, and pH (Adamczak et al., 2009).

### GOS production in a mixture of milk and permeate at various lactose conversion temperatures

**Fig. 2:**
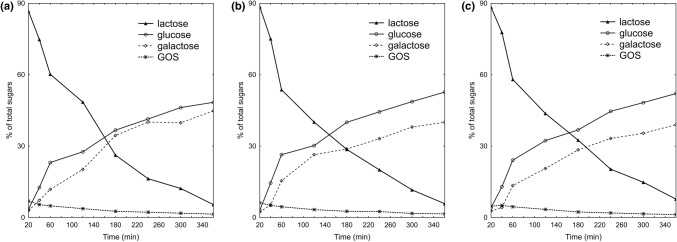
Kinetics of lactose hydrolysis and formation of galactooligosaccharides (GOS) and other sugars in permeate mixture (60:40) by using a temperature of: (**A**) 37 °C; (**B**) 40 °C; (**C**) 43 °C

**Fig. 3:**
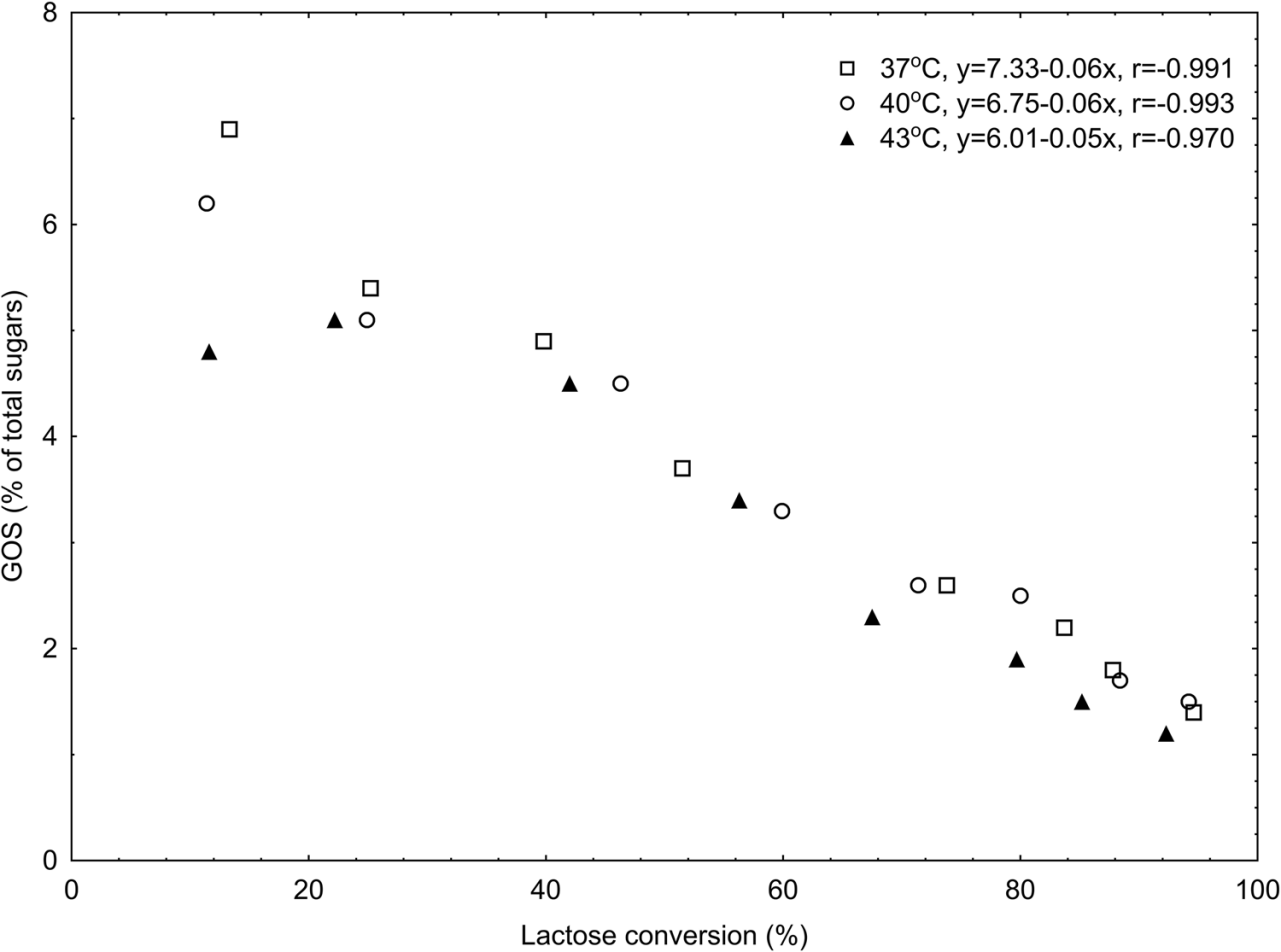
Galactooligosaccharides (GOS) production in a mixture of milk and permeate (60:40) at various lactose conversion temperatures

The proportion of GOS in the saccharide mixture was unstable and decreased with reaction time. Examining the hydrolysis and transgalactosylation of lactose in a mixture of milk and permeate (60:40) at 37 °C, 40 °C, and 43 °C, the maximum GOS content was obtained at 37 °C after 20 min (Fig. 2). It was a quantity (2.91 g/kg) that constituted 6.9% of the total sugars. At the same time, the degree of lactose hydrolysis was 13.3%. At 40 °C, the maximum content was also reached at the same time, i.e. 20 min (2.62 g/kg, which constituted 6.2% of the total sugars, the degree of lactose hydrolysis was 11.4%). However, after the same time, but at 43 °C, the content of GOS was 2.06 g/kg. The activity in this environment was shifted towards lower temperatures. The maximum level of GOS at the highest temperature was obtained after 40 min of reaction. It was a quantity of 2.15 g/kg that constituted 5.1% of the total sugars, and the degree of lactose hydrolysis was 22.2%. At the end of the reaction time (6 h) at different temperatures, the level of GOS in the total sugars in the milk and permeate mixture was about 1.4% (*p* > 0.05). At this time, at all temperatures, the degree of hydrolysis of lactose in the milk and permeate mixtures was greater than 92.3%. Analysis of DLH confirmed that the most favorable temperature for GOS synthesis was 37 °C (Fig. 3). GOS are potential substrates for lactase, which also exhibits hydrolytic activity against GOS, while GOS are created in the so-called reverse hydrolysis reaction. With progressive hydrolysis and transgalactosylation, secondary degradation of GOS results in a decrease in its level in the permeate (Demczuk et al., 2004).

For the remainder of the experiment, we selected the mixture that was formed from the bioconversion of lactose at 37 °C after 20 min. These were the conditions that guaranteed the greatest amount of resulting GOS. This model mixture of milk and permeate was further subjected to controlled fermentation to give a lactic-alcoholic product.

### Course of the fermentation process

The rate of acidification, V_m_, of the permeate and its mixture with milk was greater than that of milk alone (*p* < 0.05, Table 2). This difference deepened after the trial transgalactosylation. Permeate with GOS and its mixture (also containing GOS) reached the maximum acidification rate more rapidly (about 38% and 33%, respectively) than before the transgalactosylation. The presence of GOS also caused the permeate and its mixture to quickly reach the required acidity of pH 4.4. The time was shorter than that of milk by 11% (*p* < 0.05).

**Table 2:** Kinetic characteristics of the fermentation of goat’s milk, permeate, and its mixture before and after enzymatic hydrolysis and transgalactosylation of lactose at 37 °C, *n *= 9

Sample	*V*_*m*_ (unit pH/h)	*T*_*m*_ (h)	*T*_*e*_ (h)
*Before transgalactosylation*			
Milk	0.097a	15d	28c
Permeate	0.112c	13c	27c
Mixture 60:40	0.101b	15d	27c
*After transgalactosylation*			
Milk	0.097a	14c	27c
Permeate	0.121d	8a	23b
Mixture 60:40	0.130e	10b	24a

V_m_, maximum acidification rate; T_m_, time at which V_m_ is reached; T_e_, time to reach pH 4.45. Different small letters in superscript in columns indicate statistically significant differences at the level α = 0.05

In conclusion, this study has been shown that it is possible to produce an innovative fermented product based on goat’s milk and its MF/UF permeate. A goat’s milk/permeate mixture with a higher content of whey protein than milk could be obtained. In this mixture enzymatic hydrolysis and transgalactosylation of lactose could form GOS up to an amount equal to 7% of the total sugars. The maximum amount of GOS was accompanied by 13% lactose hydrolysis. The highest level of GOS occurred during the first 20 min at 37 °C. The mixture can be subjected to milk-alcohol fermentation. The speed of acidification of the mixture was greater than that of milk and reached the required value of pH 4.4 more quickly.
